# Spontaneous Formation of Fractal Aggregates of Au Nanoparticles in Epoxy-Siloxane Films and Their Application as Substrates for NIR Surface Enhanced Raman Spectroscopy

**DOI:** 10.3390/polym9100507

**Published:** 2017-10-13

**Authors:** Dinesh K. Basker, Kalaichelvi Saravanamuttu

**Affiliations:** Department of Chemistry and Chemical Biology, McMaster University, 1280 Main St. West, Hamilton, ON L8S 4M1, Canada; kumard3@mcmaster.ca

**Keywords:** Surface Enhanced Raman Spectroscopy (SERS), metallodielectric films, fractals, Au nanoparticles, NIR

## Abstract

We present a facile, inexpensive route to free-standing, thermo-mechanically robust and flexible epoxy-siloxane substrates embedded with fractal aggregates of Au nanoparticles, and demonstrate their efficiency as substrates for surface enhanced Raman spectroscopy (SERS) at NIR wavelengths. The metallodielectric films are prepared by generating Au nanoparticles through the in-situ reduction of gold (III) chloride trihydrate in epoxypropoxypropyl terminated polydimethyl siloxane (EDMS). The metal nanoparticles spontaneously aggregate into fractal structures in the colloid, which could then be drop-cast onto a substrate. Subsequent UV-initiated cationic polymerization of epoxide moieties in EDMS transforms the fluid colloid into a thin, free-standing film, which contains a dense distribution of fractal aggregates of Au nanoparticles. We used electron and optical microscopy as well as UV–Vis–NIR spectrometry to monitor the evolution of nanoparticles and to optically and structurally characterize the resulting films. Raman spectroscopy of the chromophore Eosin Y adsorbed onto the metallodielectric films showed that they are excellent SERS substrates at NIR excitation with an enhancement factor of ~9.3 × 10^3^.

## 1. Introduction

Raman scatter originating from the inelastic collisions between photons and molecules reveal highly specific, vibrational signatures of molecules [1,2]. The characteristically weak scattering cross-sections (typically ranging from 10^−35^ cm^2^ to 10^−25^ cm^2^ per molecule) of this spectroscopic technique [3] can be enhanced by several orders of magnitudes to 10^−17^ cm^2^ to 10^−16^ cm^2^ per molecule through Surface enhanced Raman spectroscopy (SERS) [4,5]. Such enhancement is possible when scattering occurs in the vicinity of roughened metallic surfaces or nanoparticles [6,7,8]. Here, the optical field excites surface plasmons—considered to be collective oscillations of conduction band electrons [9]—which, in turn, greatly enhance the electromagnetic field experienced by adsorbed analyte molecules [3,10,11,12,13,14]. The resulting combination of molecular specificity with high levels of sensitivity is powerful and has been exploited in SERS-based applications ranging from biomedical systems to industrial processes [10,15,16,17,18,19,20,21].

Theoretical and experimental studies have shown that the enhancement of the electromagnetic field is particularly significant at the junctions of nanoparticles, and therefore, in nanoparticle clusters and aggregates when compared to individual nanoparticles [4,22,23,24,25,26,27,28]. Placing nanoparticles in close proximity (~1 nm) causes the coupling of their dipoles; interference of coupled dipoles in turn can enhance the electromagnetic field up to 10^10^-fold [25]. Brus et al. demonstrated single molecule detection through SERS at the junction between two silver nanoparticles [24], while Xu et al. estimated an effective electromagnetic field enhancement factor of 10^10^ in the interstices of silver nanospheres [25]. Nanoparticle aggregates with fractal structure show exceptional SERS enhancement [26,29,30,31,32,33,34,35,36,37,38,39,40]; plasmonic waves become localized within fractals, which due to their lack of translational invariance cannot support ordinary wave propagation [40,41]. Because long-range interactions are suppressed in this way, individual dipolar modes of metallic particles become spatially localized and enhance the field within these nanoscopic regions. When compared to non-fractal aggregates, where the surface plasmon modes are delocalized over a longer range, electromagnetic field enhancement in fractal nanoparticle aggregates can be greater by an order of magnitude [38]. Furthermore, fractal aggregates can possess dipolar modes, which range over a wide spectral range (400 to 1750 nm), enabling surface plasmon resonance in the near-IR region [40,42,43].

Significant effort has been devoted to fabricating fractal metal structures [33,39,44,45,46,47,48] and understanding their field enhancement [26,40,41,42,49] for practical applications. For example, fractal substrates would be important in SERS-based detection techniques of biomolecules in the NIR where tissues show minimal absorbance [50,51,52]. However, it remains experimentally difficult to inexpensively generate NIR SERS substrates in the form of fractal metal nanoparticles embedded in transparent, flexible films. Such metallodielectric films could be easily integrated with sensing device architectures that are disposable and rapidly replaceable. Films containing fractal arrays of metal nanoparticles have previously been generated through lithographic techniques [53], synthetic colloidal methods [38,54], and electrochemical reduction [47]. Drop casting techniques based on evaporation-induced self-assembly have also been employed to fabricate thin film-based SERS substrates [55,56,57].

Here, we report an especially facile, two-step, room temperature, and spontaneous route to mechanically robust, flexible, optically transparent, and free-standing polymer films embedded with fractal aggregates of Au nanoparticles. Our technique involves in situ reduction of Au salts to elemental Au nanoparticles in a fluid epoxy–siloxane medium; fractal aggregates of Au nanoparticles form spontaneously upon drop-casting of the solution. They become permanently immobilized in the matrix upon photoinitiated cationic polymerization of epoxide moieties, which transforms the fluid into a thin film. We examine the microstructure of these metallodielectric films and study their effectiveness as NIR SERS substrates.

## 2. Experimental

### 2.1. Preparation of Polymer Films Doped with Au Nanoparticle Aggregates

**Scheme 1:**
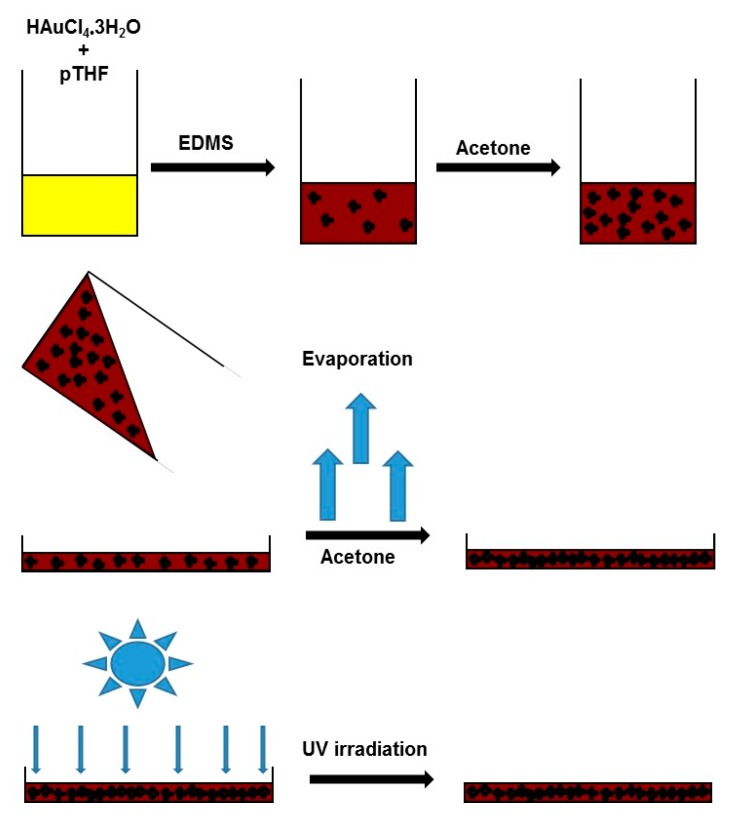
Preparation of polymer films doped with Au nanoparticle aggregates.

0.03 g (7.4 × 10^−5^ mol) of gold (III) chloride trihydrate (HAuCl_4_·3H_2_O) (Sigma-Aldrich, Oakville, ON, Canada) dissolved in 0.5 g (1.7 × 10^−4^ mol) of polytetrahydrofuran (Sigma-Aldrich, Oakville, ON, Canada) was mixed with 4.375 g (1.2 × 10^−2^ mol) of epoxypropoxypropyl terminated polydimethyl siloxane (EDMS) (Gelest, Inc., Morrisville, PA, USA) and 0.1 g (1.6 × 10^−4^ mol) of bis (4-*tert*-butylphenyl) iodoniumhexafluroantimonate (Hampford Research Inc., Stratford, CT, USA). The solution was stirred for 8 h. under minimal light exposure. The reduction of HAuCl_4_·3H_2_O by EDMS resulted in the formation of aggregates of Au nanoparticles. Free standing polymer films doped with Au nanoparticles were prepared as follows (Scheme 1): 2 g of EDMS containing Au nanoparticle aggregates was mixed with 7.5 mL of acetone and drop-cast onto a glass substrate, air dried for 1 h, and irradiated with UV light (λ = 254 nm; 26 mW) for 3 h. The resulting thin film was manually peeled off the glass substrate.

### 2.2. Characterization Techniques

UV–Vis–NIR absorbance spectra of polymer films doped with Au nanoparticle aggregates supported on glass substrates were acquired with a Cary 5000 UV–Vis–NIR spectrophotometer (Santa Clara, CA, USA). Optical micrographs were acquired in transmission mode with an Olympus BX51 microscope (Tokyo, Japan) fitted with a Q-Imaging Retiga EXi digital camera and ImagePro. software (Surrey, BC, Canada) Scanning electron micrographs (SEM,) were recorded with a VEGA3 SB instrument (Brno, Czech Republic) with an accelerating voltage of 10 kV in back-scattering mode. Transmission electron microscopy (TEM) and energy dispersive X-ray spectrometry (EDX) were carried out with a Philips CM12 instrument (Amsterdam, The Netherlands) at an accelerating voltage of 200 kV. Samples for SEM and TEM from thin films were ultra-microtomed under cryogenic conditions. TEM samples of Au nanoparticles (after 30 min of stirring) and aggregates (after 8 h of stirring) were prepared by diluting respective colloids in acetone and drop-casting onto Cu grids.

### 2.3. Measurement of Fractal Dimension

We used the box counting method [58] to obtain the Hausdroff dimension (*D*) or the fractal dimension. In this method, a grid of perpendicular lines separated by distance ϵ is first superimposed onto the image of the aggregate structure. Then, the number of grid boxes (*n*) containing the aggregates (and leaving behind grid boxes with empty space) is counted. The slope of the square linear fit of log *n* verses log (ϵ) yields *D*.

*D =* log *n/*log *ϵ·lim ϵ → 0*

We used the ImageJ plugin FracLac (NIH, Bethesda, USA) [58] to execute the box counting method. FracLac was first verified using the Sierpinski triangle, a known fractal structure, which yielded *D* = 1.535; the corresponding error is 3% (*D* for Sierpinski triangle = 1.585). Optical micrographs were acquired over areas of ≈0.15 mm^2^ of the thin films embedded with Au nanoparticle aggregates. The micrographs were then converted into binary images through ImageJ before the FracLac procedure was applied. A sample calculation is provided as Supplementary Information (Figure S1, Supplementary Materials).

### 2.4. SERS Experiments

Eosin Y (2′,4′,5′,7′-tetrabromo-3′,6′-dihrdroxyspiro[2-benzofuran-3,9′-xanthene]-1-one) (99%; Sigma Aldrich, Oakville, ON, Canada) was used without further purification. 50 μL of a 1 mM solution of Eosin Y in methanol was spread homogeneously on a polymer film containing Au nanoparticle aggregates and air-dried for 30 min. Raman spectra were acquired at the excitation wavelength = 785 nm with a Reinshaw Invia Laser Raman Spectrometer (~1.5 mW (10% of total power), exposure time = 10 s, Wotton-under-Edge, UK) operating in confocal mode with a 20× microscope objective; the spectral resolution was 1.0 cm^−1^. The 65 µm-width of the photodetector slit together with the laser focus depth of 1 µm was employed to determine the sample volume that was probed in the experiment. Control spectra were acquired with 1 M of dye solution coated on polymers films that did not contain Au nanoparticle aggregates; identical preparation and spectral acquisition conditions were employed.

## 3. Results and Discussion

### 3.1. Generation of Au Nanoparticles in the Epoxy Medium

**Figure 1:**
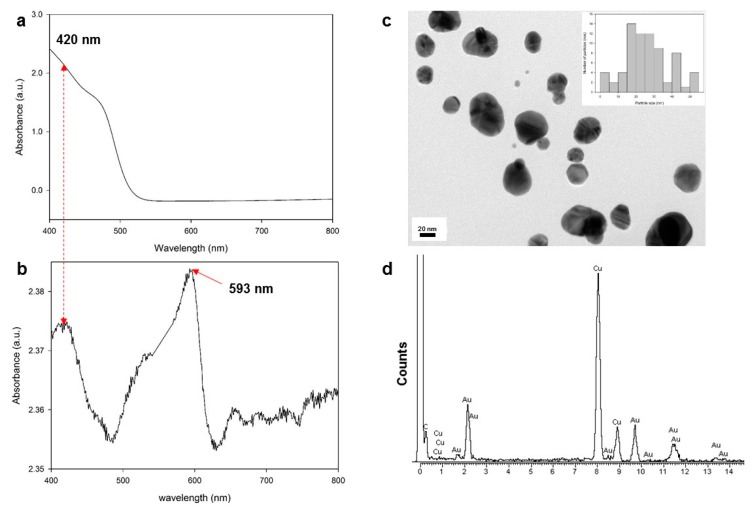
Au nanoparticles formed through reduction of Au(III) salt by polydimethyl siloxane (EDMS) after 30 min of stirring. (**a**) UV–Vis spectrum of 7.4 × 10^−5^ mol Au salt in pTFH; (**b**) UV–Vis absorbance spectrum of Au nanoparticles after addition of EDMS (the red dotted line is included for ease of comparison between (**a**) and (**b**)); (**c**) transmission electron micrograph, inset shows corresponding size distribution (*N* = ~70 particles) and (**d**) Energy dispersive X-ray spectrum of resulting Au nanoparticles.

Au nanoparticles were generated through in-situ chemical reduction of gold (III) chloride trihydrate by epoxypropoxypropyl terminated polydimethyl siloxane (EDMS) [59,60,61]. When 7.4 × 10^−5^ mol of Au salt dissolved in 0.5 g (1.7 × 10^−4^ mol) of pTHF was mixed with EDMS and stirred for 30 min, and the originally yellow coloured Au salt solution turned deep purple upon the formation of Au nanoparticles. This was confirmed through the emergence of an absorbance band maximising at ~590 nm (Figure 1b), which can be attributed to the dipolar plasmon resonance of spherical Au nanoparticles [62]. The bathochromic shift of this band as compared to the characteristic resonance wavelength of Au nanoparticles at ~520 nm arises from the greater dielectric constant of the EDMS medium (~1.5) when compared to the typically employed H_2_O medium (~1.3) [63,64]. Transmission electron micrographs of the sol cast onto a Cu grid revealed a polydisperse population of Au nanoparticles with an average diameter of 25 ± 12 nm (Figure 1c,d). EDX carried out on the particles confirmed their composition to be elemental Au (Figure 1d).

Au(III) salt in this system is reduced by the ether moieties of EDMS [59,60,61]. Particle formation is initiated when [AuCl_4_]^−^ ions bind to pseudocrown ether cavities formed by EDMS oligomers and are reduced to Au (I) [59,60,61]. This reaction disrupts the ether cavities and releases Au (I) species, which are further reduced to Au atoms. Atomic ensembles of Au then combine to form nanoparticles through Ostwald ripening [65]. The size of individual nanoparticles formed is determined by the weight ratio of ether moieties to Au (III) ions, which controls the kinetics of reduction reactions [61].

**Figure 2:**
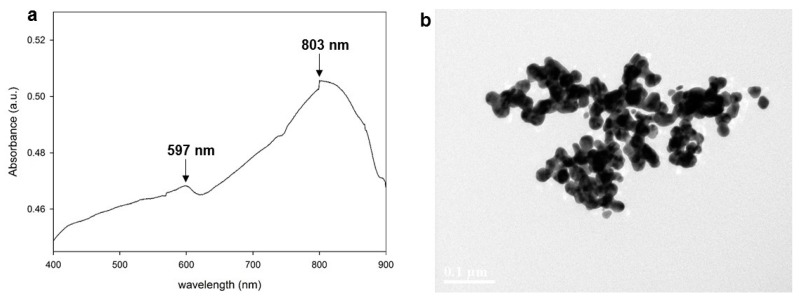
(**a**) UV–Vis–NIR spectrum and corresponding (**b**) transmission electron micrographs of Au nanoparticle aggregates in EDMS after >8 h of stirring.

Over time, the Au nanoparticles in the colloid form aggregate. Figure 2a is the UV–Vis absorbance spectrum of the colloid containing Au nanoparticles after it had been continuously stirred for >8 h. When compared to the spectrum of the colloid acquired after only 30 min of stirring (Figure 1b), there is a relative decrease in absorbance at ~590 nm and a relative increase in the band maximizing at ~800 nm. Transmission electron micrograph of the colloid revealed the presence of nanoparticle aggregates (Figure 2b) in contrast to the discrete particles present in samples stirred for only 30 min (Figure 1c). From transmission electron micrographs, we reason that the nanoparticle aggregates form through the diffusion limited aggregation (DLA) mechanism [66,67,68,69,70] Here, particles formed at the early stages (e.g., Figure 1c) serve as seeds, which bind to other discrete particles undergoing Brownian motion.

The UV–Vis absorbance spectrum in Figure 2a indicates that aggregates are present in the colloid (and that they do not form only when cast on TEM grids). The absorbance maximum in the visible region can be attributed to the quadrupole plasmon resonance of aggregated Au particles, whereas the band in the NIR region originates from their dipole plasmon resonance [71,72,73,74]. The bathochromic shift of the dipole resonance originates from near field coupling, which accompanies the decrease in interparticle separation [72]. This red-shifted band is relatively broad, which is also a characteristic of aggregates [40]. Similarly, the excitation of quadrupole resonance modes at visible wavelengths (lower energies) relative to single nanoparticles is characteristic of aggregate structures [72,73,74]. Importantly, the spectrum in Figure 2a is distinct from extinction spectra of asymmetric Au nanoparticles such as rods and shells [6,51,52]; here, the multiple absorbance bands arise from the excitation of transverse and longitudinal resonance modes [6,51,52].

### 3.2. Flexible, Free-Standing Thin Films Embedded with Fractal Aggregates of Au Nanoparticles

**Scheme 2:**
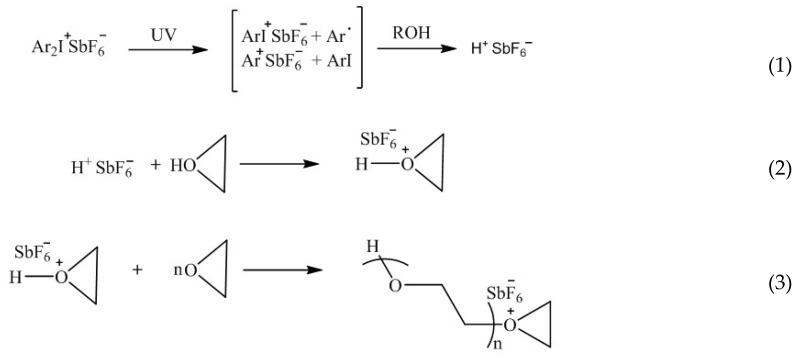
UV-initiated polymerization of epoxide groups in EDMS [75].

**Figure 3:**
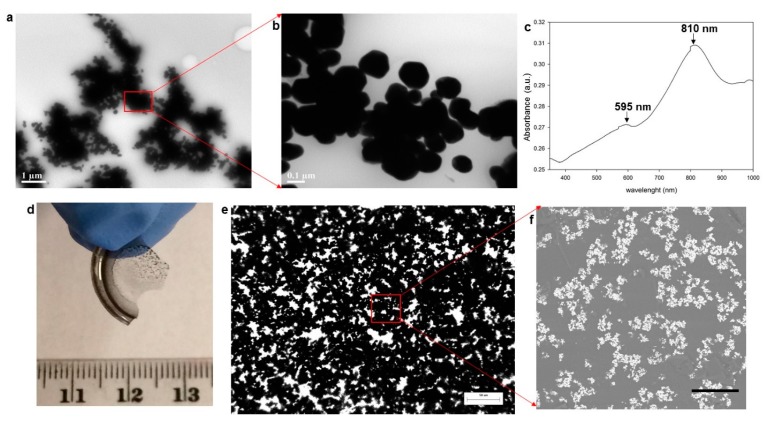
(**a**) Transmission electron micrograph; (**b**) corresponding magnified view; (**c**) UV–Vis–NIR absorbance spectrum and (**d**) photograph of free-standing epoxy thin films containing Au nanoparticle aggregates; (**e**) Optical micrograph of the film dense distribution of Au nanoparticle aggregates (scale bar = 50 μm); and, (**f**) scanning electron micrograph acquired in backscattering mode (scale bar = 20 μm).

Thin films embedded with fractal aggregates of Au nanoparticles were prepared through drop-casting. The colloid, which had been stirred for >8 h and therefore contained Au nanoparticle aggregates, was first diluted in acetone (2 g colloid in 7.5 mL of acetone) and then cast onto a glass substrate. The sample was air-dried before being irradiated with UV light, which initiated the polymerization of epoxide moieties in the medium (Scheme 2) [75]. UV irradiation of diaryl iodonium salt in the presence of the nonbasic hydrogen donor EDMS generates a Brönsted acid, which in turn initiates the ring opening polymerization of the terminal epoxide groups of EDMS. Polymerization transforms the fluid colloid into a free standing, transparent, purple-tinted film. Transmission electron microscopy confirmed the presence of aggregates composed of Au nanoparticles (Figure 3a,b). The UV–Vis–NIR absorbance spectrum of the film (Figure 3c) was comparable to the spectrum of the fluid colloid (Figure 2a), confirming that neither drop-casting nor subsequent polymerization processes significantly affect the distribution of Au nanoparticles in the colloid. Accordingly, the optical micrographs revealed the dense distribution of nanoparticle aggregates throughout the ~5 cm^2^ area of the film (Figure 3e and Figure S2, Supplementary Materials). Scanning electron micrographs acquired in backscattering mode (Figure 3f) closely matched the optical micrograph and further confirmed that the aggregates consisted of metal nanoparticles.

Aggregates of Au nanoparticles in the epoxy thin films possessed fractality, as defined by the Hausdroff dimension or fractal dimension *D*; the greater the value of *D*, the more complex the fractal structure [30]. We determined the average value of *D* for ten different optical micrographs of epoxy thin films (area = ~0.15 mm^2^) containing nanoparticle aggregates and obtained *D* = 1.801 ± 0.019, which shows the fractal nature of these aggregates [67].

### 3.3. Surface Enhanced Raman Scattering

Epoxy films doped with fractal aggregates of Au nanoparticles are efficient substrates for SERS with NIR excitation. Importantly, the fractal arrangement of nanoparticle aggregates is expected to significantly enhance the SERS response as compared to systems without fractality [31,32,40,41]. The enhancement in fractal systems, which lack translational invariance and therefore do not support propagating surface plasmon modes, originates from localized modes [40,41]. This is different from the enhancement observed in periodic nanostructures such as nanoparticle (spherical, rod, and cubic) arrays, nanometallic gratings and nanohole arrays, which originates predominately from propagating surface plasmons. Surface plasmon modes in fractals originate from dipole-dipole coupling interactions of individual metal nanoparticles [40,41]. The resulting modes are tightly confined, giant fluctuations of electric field; the local field enhancement in these “hotspots” can exceed 10^3^-fold relative to comparable systems that do not possess fractality [30,31,32,40,41].

**Figure 4:**
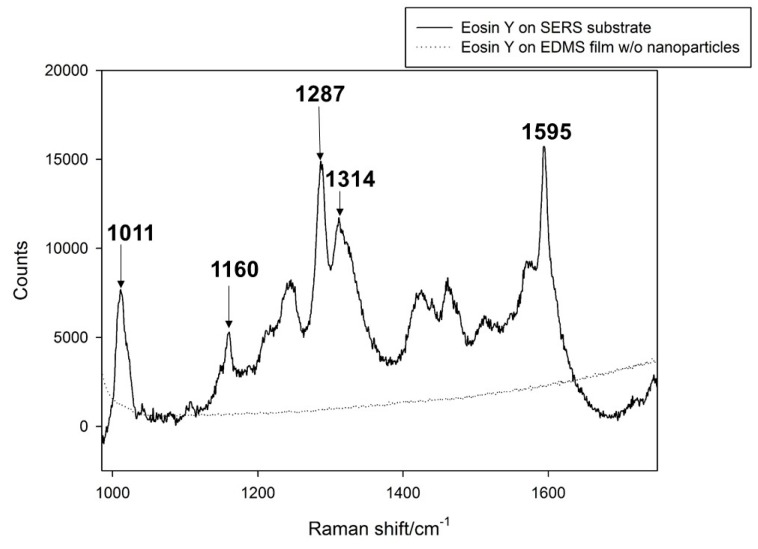
Raman spectra of Eosin Y (1 mM) adsorbed on epoxy film containing fractal aggregates of Au nanoparticles Eosin Y (1 M) on epoxy film without nanoparticles. The same acquisition conditions were used to acquire both spectra.

We demonstrated SERS with NIR excitation of a standard dye Eosin Y adsorbed on an epoxy film doped with fractal aggregates of Au nanoparticles [76]. Figure 4 is the SERS spectrum of a 1 mM solution of Eosin Y adsorbed on the thin metallodielectric film (Figure S3); a control spectrum acquired under identical conditions of 1 M solution on an epoxy thin film that did not contain aggregates of Au nanoparticles is included for comparison. Peaks assigned to the Raman modes of Eosin Y (Table 1) [77] are significantly enhanced in the case of epoxy substrates containing fractal aggregates of Au nanoparticles when compared to substrates without nanoparticles, in which the signals are negligible. To quantify the enhancement, we compared the intensity of the xanthane and benzene ring stretching modes (1310 cm^−1^) for the metallodielectric substrate (*I*_SERS_) and the control substrate (*I*_NOR_) according to [78]:

Enhancement Factor = (*I*_SERS_*N_NOR_)/(*I*_NOR_*N_SERS_) (4)

Here, *N*_SERS_ and *N*_NOR_ correspond to the approximate number of Eosin Y molecules adsorbed on each type of substrate. These values were calculated by taking into account the concentration of Eosin Y and approximating the probed volume to be a cylinder [78], using the 65 µm-width of the photodetector slit together with the laser focus depth of 1 µm. The resulting values of *N*_NOR_ = 1.9 × 10^12^ [15] and *N*_SERS_ = 1.9 × 10^9^ yielded an enhancement factor of 9.3 × 10^3^ (because electronic excitations at NIR wavelengths is negligible for Eosin Y, we reason that there is no contribution from resonance Raman scattering here).

## 4. Summary and Outlook

We have demonstrated an inexpensive and spontaneous route to generate fractal aggregates of metal nanoparticles embedded in a free-standing polymer film, which can serve as efficient SERS substrates at NIR wavelengths. Although SERS enhancement factors greater by up to four orders of magnitude are possible through other fractal Au nanostructures [30,38], these systems are typically fabricated through complex routes such as electron beam lithography [53], synthetic colloidal techniques [38,54], and electrochemical reduction [47]. The resulting systems are expensive and often structurally rigid, making their implementation in high throughput, practical applications difficult. While drop casting alone has been elegantly exploited to generate metal nanoparticle patterns in polymer SERS substrates [55,56,57], our approach combines photopolymerization of the polymer host with drop-casting to generating free-standing substrates. Moreover, thin films generated by drop casting is usually limited to thermoplastic polymers or linear polymers as it requires the dissolution of polymer in a suitable solvent carrier [55,57]. By contrast, our approach extends this technique to thermosetting polymers like epoxides by first drop-casting the monomer (which is soluble in polar solvent unlike the corresponding polymer) containing Au metal nanoparticles and then generating the film through cationic polymerization. Polymerization permanently locks the fractal nanoparticle aggregates into place. The resulting SERS substrates, which are based on an epoxide–siloxane matrix, are thermo-mechanically stable and possess good solvent resistance [79]. While a film comprising of epoxy alone would be rigid, the siloxane component in our system imparts flexibility to the thin film without compromising its transparency or thermo-mechanical stability (Figure S4, Supplementary Materials). The medium swells in solvents, which would allow analyte solutions to permeate the matrix and access hot spots generated by the fractal aggregates of metal nanoparticles. Additionally, the excellent transparency of the epoxy–siloxane matrix in the NIR region minimizes attenuation of the probe laser beam, enabling it to efficiently excite the SPR of Au nanoparticles.

## Figures and Tables

**Table 1:** Calculated and experimental Raman scattering modes of Eosin Y at 785 nm excitation [77].

Shifted Calculated Mode cm^−1^	Description	Experimental Shift cm^−1^
1003	xanthene ring breathing + C−Br stretches	1011
1158	xanthene and benzene ring stretches and symmetric CO_2_ stretch	1160
1260	xanthene and benzene ring C−C stretches	1287
1310	xanthene and benzene ring stretches and symmetric CO_2_ stretch	1314
1579	xanthene and benzene ring stretches and symmetric CO_2_ stretch	1579

## Supplementary Materials

The following are available online at www.mdpi.com/2073-4360/9/10/507/s1, Figure S1: Determination of fractal dimension; Figure S2: optical micrographs of epoxide films containing aggregates of Au nanoparticles; Figure S3: SERS spectra of 1 mM solution of Eosin Y reproduced on 3 different samples of epoxide films containing fractal aggregates of Au nanoparticles; Figure S4: Photographs of free-standing epoxide film containing aggregates of Au nanoparticles.
