# Supplementary material for: Spontaneous Formation of Fractal Aggregates of Au Nanoparticles in Epoxy-Siloxane Films and Their Application as Substrates for NIR Surface Enhanced Raman Spectroscopy
Source: Polymers (Basel). 2017 Oct 13;9(10):507. doi: 10.3390/polym9100507 (PMC6418541; doi:10.3390/polym9100507)
Supplement: Supplementary file 1 [file polymers-09-00507-s001.pdf]

Supplementary information

Spontaneous formation of fractal aggregates of Au nanoparticles in epoxy-siloxane films and their application as substrates for NIR Surface Enhanced Raman Spectroscopy

Dinesh K. Basker and Kalaichelvi Saravanamuttu*

Department of Chemistry and Chemical Biology, McMaster University, 1280 Main St. West, Hamilton, Ontario L8S 4M1, CANADA

Corresponding author:
Dr. Kalaichelvi Saravanamuttu,
Associate Professor,
Department of Chemistry and Chemical Biology,
McMaster University
1280 Main St. West, L8S 4M1,
Ontario, CANADA
E-mail: kalai@mcmaster.ca

Measurement of fractal dimension D

D was determined through the box counting method.[S1] In this method, D is measured by placing several grids of decreasing box size and by counting the number of boxes containing the fractal structure. The slope of the log-log plot of number of boxes containing the parts of fractal structure (n, count) *versus* size of the box (ε), gives the fractal dimension D.

$$D = \log n / \log \epsilon \quad \lim \epsilon \rightarrow 0$$

Measurements were carried out on binary images of optical micrographs. A sample procedure is shown in Figure S3; first, the optical micrograph (Figure S3(a)) is binarized (Figure S3(b)) before applying the box counting method (using ImageJ plugin fraclac). A log-log plot of n versus ε is then generated (Figure S3c), the slope of which yields the fractal dimension.

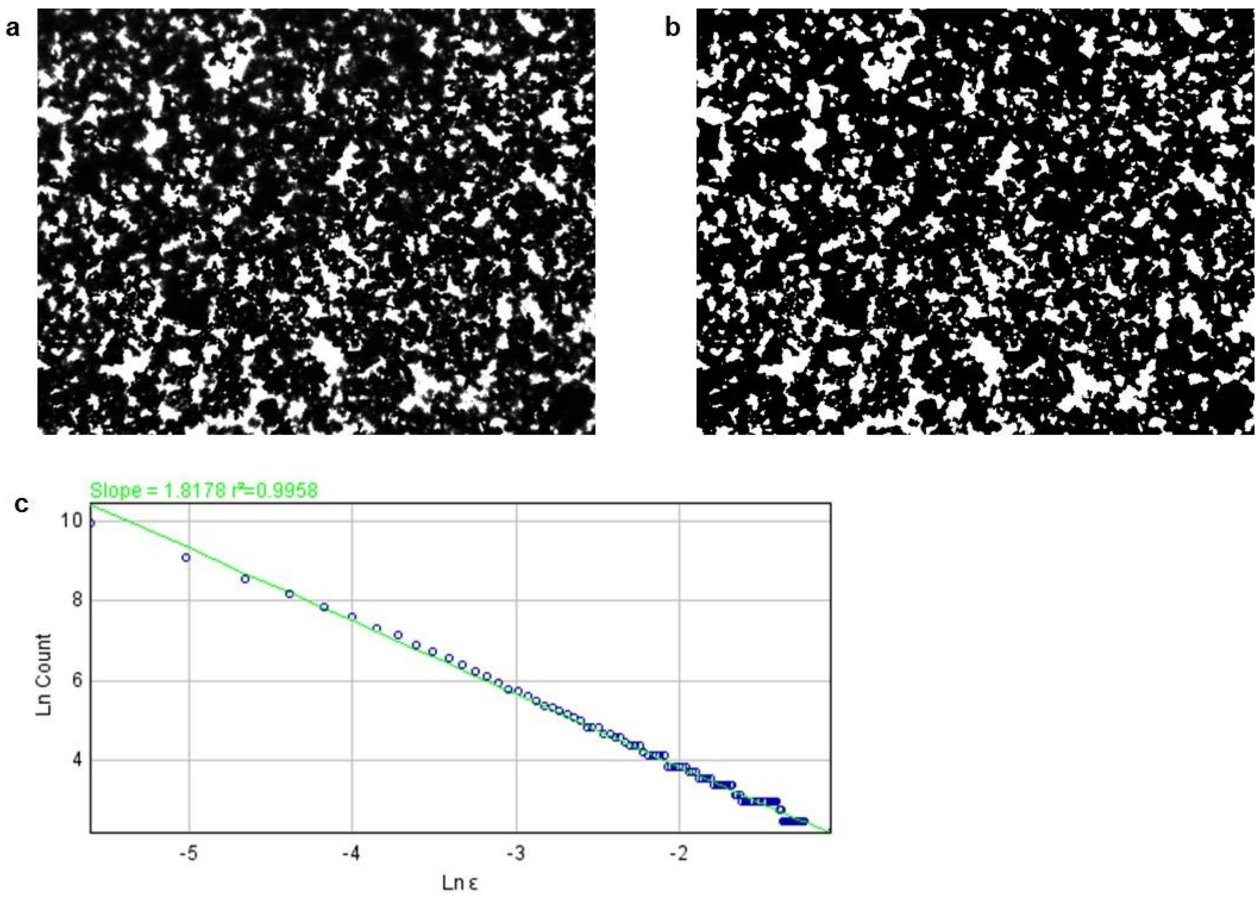

Figure S1. Determination of fractal dimension using box counting method (a) optical micrograph of thin film embedded with Au nanoparticles fractal aggregates (b) corresponding binary image (c) log-log plot of n *vs* ε, with the slope giving the fractal dimension

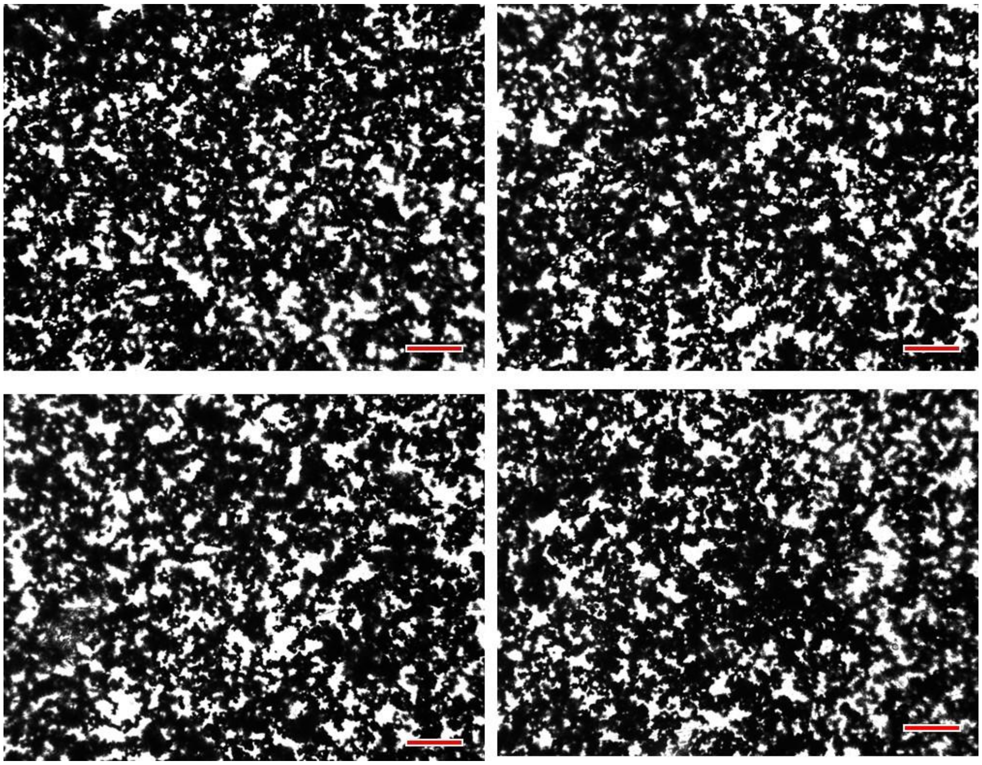

Figure S2. Examples of optical micrographs of epoxide films containing aggregates of Au nanoparticles employed to determine the fractal dimension of aggregates (scale bar = 50 μm)

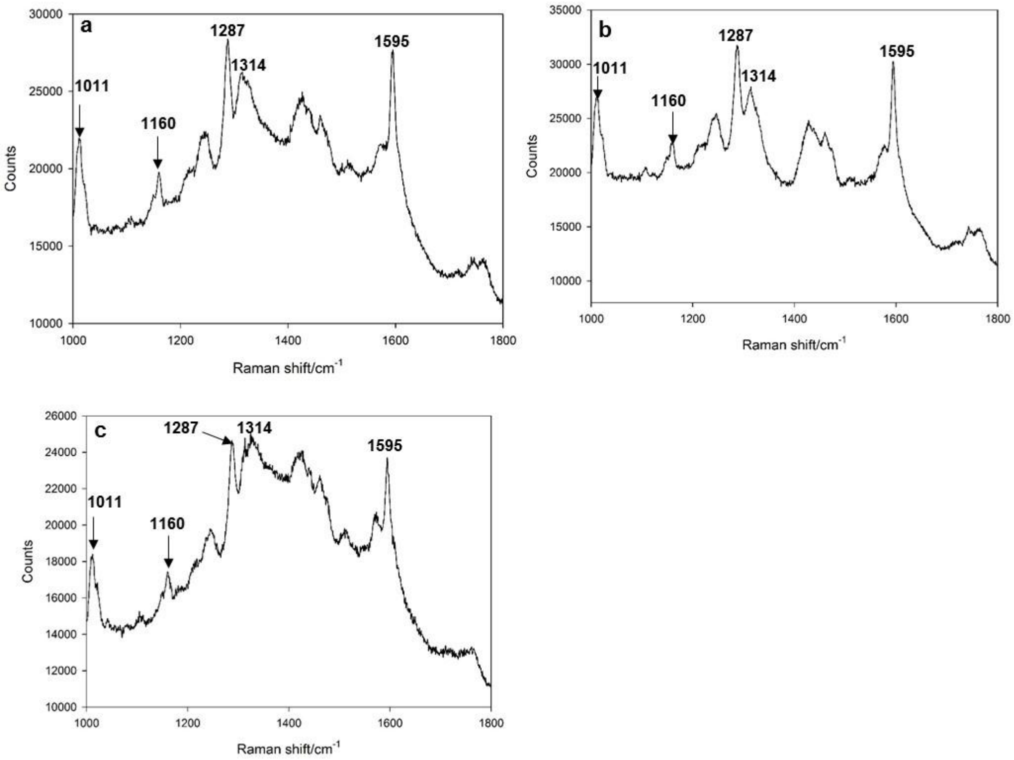

Figure S3. SERS spectra of 1 mM solution of Eosin Y reproduced on 3 different samples of epoxide films containing fractal aggregates of Au nanoparticles. All films were prepared under identical conditions and all spectra were acquired with the same parameters.

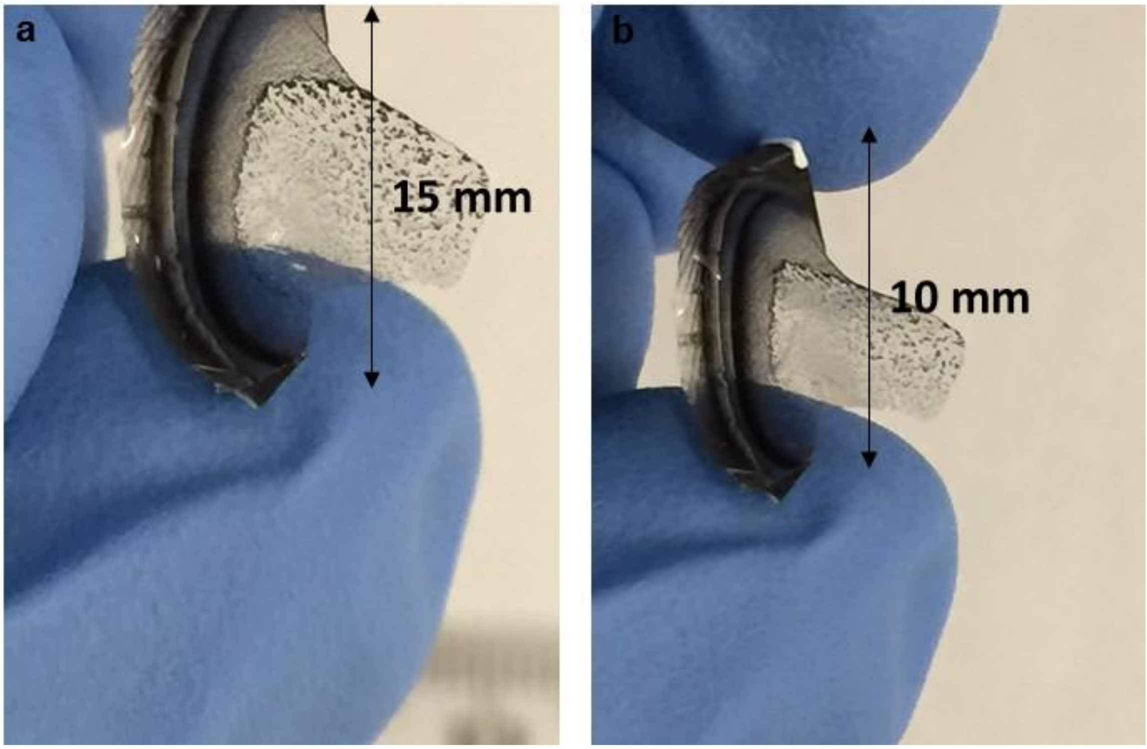

Figure S4. Photographs of free-standing epoxide film containing aggregates of Au nanoparticles in (a) normal and (b) flexed position.

Reference

S1. A. Hecht, P. Commiskey, F. Lazaridis, P. Argyrakis and R. Kopelman, *ChemPhysChem*, **2014**, *15*, 3444–3446.
